# Supplementary material for: Demographic, socioeconomic and regional disparities in the coverage of water, sanitation and hygiene facilities in four South Asian Countries
Source: PLoS One. 2025 Mar 17;20(3):e0319754. doi: 10.1371/journal.pone.0319754 (PMC11913270; doi:10.1371/journal.pone.0319754)
Supplement: S1 Table — (DOCX) [file pone.0319754.s001.docx]

S1 Table: GVIF for binary logistic regression model adjusted for demographic, socio-economic, and geographic factors with WASH facilities as outcome in Afghanistan and Bangladesh.

|  | **Afghanistan** (2022) | | | | **Bangladesh** (2019) | | | |
| --- | --- | --- | --- | --- | --- | --- | --- | --- |
| **Variables** | **GVIF** | **Df** | **Adjusted GVIF** | **Squared Adjusted GVIF** | **GVIF** | **Df** | **Adjusted GVIF** | **Squared Adjusted GVIF** |
| **Place of residence** | 1.58 | 1 | 1.26 | 1.58 | 1.08 | 1 | 1.04 | 1.08 |
| **Economic status** | 1.81 | 2 | 1.16 | 1.35 | 1.71 | 2 | 1.14 | 1.31 |
| **Sex of household head** | 1.1 | 1 | 1.05 | 1.1 | 1.05 | 1 | 1.02 | 1.05 |
| **Age of household head** | 1.42 | 2 | 1.09 | 1.19 | 1.18 | 2 | 1.04 | 1.09 |
| **Education of household head** | 1.38 | 3 | 1.06 | 1.11 | 1.37 | 3 | 1.05 | 1.11 |
| **Religion of household head** | - | - | - | - | 2.79 | 2 | 1.29 | 1.67 |
| **Ethnicity** | - | - | - | - | 1.14 | 1 | 1.61 | 2.59 |
| **Family size** | 1.42 | 2 | 1.09 | 1.19 | 1.35 | 2 | 1.03 | 1.07 |
| **Mass media accessibility** | 1.27 | 1 | 1.13 | 1.27 | 1.47 | 1 | 1.16 | 1.35 |
| **Region** | 2.2 | 7 | 1.06 | 1.12 | 2.59 | 7 | 1.03 | 1.06 |
