# Supplementary material for: Demographic, socioeconomic and regional disparities in the coverage of water, sanitation and hygiene facilities in four South Asian Countries
Source: PLoS One. 2025 Mar 17;20(3):e0319754. doi: 10.1371/journal.pone.0319754 (PMC11913270; doi:10.1371/journal.pone.0319754)
Supplement: S2 Table — (DOCX) [file pone.0319754.s002.docx]

S2 Table: GVIF for binary logistic regression model adjusted for demographic, socio-economic, and geographic factors with WASH facilities as outcome in Nepal and Pakistan.

|  | **Nepal** (2019) | | | | **Pakistan** (2017-19) | | | |
| --- | --- | --- | --- | --- | --- | --- | --- | --- |
| **Variables** | **GVIF** | **Df** | **Adjusted GVIF** | **Squared Adjusted GVIF** | **GVIF** | **Df** | **Adjusted GVIF** | **Squared Adjusted GVIF** |
| **Place of residence** | 1.2 | 1 | 1.09 | 1.2 | 1.59 | 1 | 1.26 | 1.59 |
| **Economic status** | 1.69 | 2 | 1.14 | 1.3 | 1.72 | 2 | 1.14 | 1.31 |
| **Sex of household head** | 1.38 | 1 | 1.17 | 1.38 | 1.13 | 1 | 1.06 | 1.13 |
| **Age of household head** | 1.5 | 2 | 1.11 | 1.23 | 1.15 | 2 | 1.04 | 1.07 |
| **Education of household head** | 1.65 | 3 | 1.09 | 1.18 | 1.28 | 3 | 1.04 | 1.09 |
| **Religion of household head** | 1.24 | 2 | 1.06 | 1.12 | - | - | - | - |
| **Ethnicity** | - | - | - | - | - | - | - | - |
| **Family size** | 1.29 | 2 | 1.07 | 1.14 | 1.14 | 2 | 1.03 | 1.07 |
| **Mass media accessibility** | 1.2 | 1 | 1.1 | 1.2 | 1.09 | 1 | 1.05 | 1.09 |
| **Region** | 1.85 | 6 | 1.05 | 1.11 | 1.38 | 3 | 1.06 | 1.11 |
