# Supplementary material for: Demographic, socioeconomic and regional disparities in the coverage of water, sanitation and hygiene facilities in four South Asian Countries
Source: PLoS One. 2025 Mar 17;20(3):e0319754. doi: 10.1371/journal.pone.0319754 (PMC11913270; doi:10.1371/journal.pone.0319754)
Supplement: Attachment — Submitted filename: Comments.docx [file pone.0319754.s003.docx]

Title: **Demographic, Socioeconomic and Regional Disparities in the Coverage of Water, Sanitation and Hygiene (WASH) Facilities in Four South Asian Countries**

Authors: Md. Hasibul Islam Jitu & Mohammad Shahed Masud

Objective: The main objective of this study was to (i)review the existing WASH facilities, (ii) mapping for regional comparisons, and (iii)identify the significant socioeconomic and demographic factors associated with WASH facilities in four South Asian countries: Bangladesh, Pakistan, Nepal, and Afghanistan.

The authors have tried to explore the situation of WASH facilities in four SAARC countries.

I have some observations on the paper and hope that this will add value to paper.

1. Abstract (Background): Add the MICS date right after the country in the bracket [such as Nepal (MICS 2019)]. Could you please present the names of countries in ascending (a to z) order, i.e., Afghanistan, Bangladesh, Nepal, and Pakistan, throughout the text? Please ensure discussion in that way. Abstract (Results): Add the value of a particular variable in the results so that the reader may understand the magnitude of the influence of variables.
2. Abstract (Conclusion): Suggest specific recommendations rather than general ones. How could policymakers use your finding into practice/intervention planning?
3. Follow the format and guidelines of PLoS One.
4. Did you get permission to use the datasets of MICS since you are going to publish them from concerned authorities?
5. In Methods: Covariates section, what is the logic for categorizing the age group <35, 35-54, and 54+, in ethnicity Bengali and others? Please present it in a logical order. For ethnicity, it may be indigenous, and others might be appropriate for all countries and categorize them accordingly. In the same way, how did you categorize poor, middle, and rich instead of five quintiles?
6. Ethical consideration: Please provide the date of ethical approval and the authority that provided the ethical clearance country-wise.
7. Please provide the date of the survey along with the country in brackets in all tables, such as Nepal (2019).
8. The authors have presented the exact description of the table. I would recommend presenting the impression of the table rather than the exact numeric value.
9. Did you assess the multi-collinearity issue before performing multivariate analysis?
10. Why and how did you claim that 'the model has quite well discriminating ability' and present it with logic and reference (citation)?
11. In the discussion session, I would recommend linking with theories related to WASH. Moreover, discuss with Sustainable WASH principles: technical, institutional, environmental, financial, and social sustainability of WASH.
12. Add the strengths and limitations of the study before the conclusion session.
13. Consult with recent and current literature.
14. Go through the text for grammar, language, and coherence.
